# Characterization of Emerging Swine Viral Diseases through Oxford Nanopore Sequencing Using Senecavirus A as a Model

**DOI:** 10.3390/v12101136

**Published:** 2020-10-07

**Authors:** Shaoyuan Tan, Cheryl M. T. Dvorak, Michael P. Murtaugh

**Affiliations:** Department of Veterinary and Biomedical Sciences, College of Veterinary Medicine, University of Minnesota, St. Paul, MN 55108, USA; tanxx606@umn.edu

**Keywords:** emerging infectious diseases, molecular diagnostic techniques, Oxford Nanopore sequencing, Senecavirus A, bioinformatics, swine viral diseases

## Abstract

Emerging viral infectious diseases present a major threat to the global swine industry. Since 2015, Senecavirus A (SVA) has been identified as a cause of vesicular disease in different countries and is considered an emerging disease. Despite the growing concern about SVA, there is a lack of preventive and diagnostic strategies, which is also a problem for all emerging infectious diseases. Using SVA as a model, we demonstrated that Oxford Nanopore MinION sequencing could be used as a robust tool for the investigation and surveillance of emerging viral diseases. Our results identified that MinION sequencing allowed for rapid, unbiased pathogen detection at the species and strain level for clinical cases. SVA whole genome sequences were generated using both direct RNA sequencing and PCR-cDNA sequencing methods, with an optimized consensus accuracy of 94% and 99%, respectively. The advantages of direct RNA sequencing lie in its shorter turnaround time, higher analytical sensitivity and its quantitative relationship between input RNA and output sequencing reads, while PCR-cDNA sequencing excelled at creating highly accurate sequences. This study developed whole genome sequencing methods to facilitate the control of SVA and provide a reference for the timely detection and prevention of other emerging infectious diseases.

## 1. Introduction

Emerging and re-emerging viral diseases have had a significant adverse impact on swine production and will be an ongoing challenge for the swine industry. Emerging infections can be caused by previously unknown/undetected agents or known pathogens spreading to a new geographic location or host. The appearance of emerging diseases has usually been characterized by sudden unpredictable outbreaks, which then spread across regions and countries [1]. This feature drives the need for effective emerging disease management via robust pathogen detection (including novel pathogens) and efficient epidemiological surveillance [2,3,4]. 

Over the last 40 years, emerging pathogens that cause devastating swine diseases include porcine reproductive and respiratory syndrome virus (PRRSV) first described in the late 1980s [5], porcine circovirus type 2 (PCV2) discovered in the late 1990s [6] and more recent porcine epidemic diarrhea virus (PEDV) appearing in the US in the early 2010s [7]. The current introduction of African Swine Fever (ASF) into China, affecting over half of China’s swine herds, confirms the significant impact that viral diseases have on the swine industry. To date, ASF has spread from China to neighboring countries and it is very likely to eventually enter other ASF-free regions, such as the United States, despite all attempts to keep it out [8]. The United States is now free of foot-and-mouth disease (FMD), ASF, and classical swine fever (CSF), but these diseases are very likely to eventually arrive in the US due to increased travel and trade activities.

Senecavirus A (SVA), is a single stranded, positive-sense RNA virus of approximately 7.3 kilobases belonging to the family *Picornaviridae*, genus *Senecavirus* [9,10]. Previously, the study of SVA mainly focused on its use for oncolytic virotherapy [11]. Research into the control of SVA in swine is now under examination since SVA has been detected in association with outbreaks of vesicular disease and neonatal mortality in pigs worldwide since 2015, including the US, Brazil, Thailand and China [12,13,14,15]. Due to its rapid spread, SVA is considered an emerging infectious disease [16]. To date, the risk of SVA causing an epidemic remains a possibility and the better control of SVA to minimize viral spread needs to occur. 

Effective surveillance and rapid response to SVA infections are imperative to reduce its adverse impact on the swine industry, which depends on the development of diagnostic tools. A variety of diagnostic methods have been developed, including virus isolation, direct detection in tissues by immunohistochemistry, serological assays such as ELISA and virus neutralization, and nucleic acid detection using PCR [12,17,18,19,20,21,22]. The methods mentioned above play an important role in SVA detection, however, they are all hypothesis-driven methods which require prior information about the pathogen in order to detect it. Thus, when dealing with an unexpected emerging virus or mutated strains at early stages, where little or no information is present, these methods will inevitably slow down the pathogen detection process. High-throughput sequencing technologies have brought a new opportunity for the surveillance and diagnostics of emerging viruses [23]. The robust, unbiased features of sequencing allow for the timely discovery of unexpected and even novel pathogens. The genomic information generated from sequencing can be used to support the development of other diagnostic methods, such as primer design for PCR. Additionally, the subsequent whole genome generation from sequencing can enlarge the genome database to aid in the further investigation of disease mechanisms or epidemiological investigations [24,25]. This is beneficial in the case of SVA, since very few SVA whole genomes are available in the NCBI GenBank resource (i.e., 203 complete genomes as of Jan. 2020). As more SVA genomes are sequenced and analyzed, our understanding of SVAs will improve, including SVA genotyping, molecular epidemiology, disease transmission mechanisms, and vaccine development. 

Oxford Nanopore Technologies (ONT) MinION (ONT, Oxford, UK) is a single-molecule, long-read sequencer that determines bases by detecting ionic current changes as a DNA or RNA strand passes through a nanoscale protein pore [26]. The MinION sequencer is highly accessible to any research group or diagnostic laboratory due to its low capital investment, low maintenance and easy operation. It is portable (the size is smaller than a smart phone) and thus is potentially able to fulfill a point-of-care test in field environments [27]. MinION sequencing has already been used for in-field pathogen diagnostics including Ebola, Zika and influenza surveillance and outbreak investigations [28,29,30]. This study evaluated and compared Oxford Nanopore direct RNA sequencing (DRS) and PCR-cDNA sequencing (PCS) for rapid detection and genome generation of RNA viruses using SVA as a model. SVA was successfully detected at the species and strain level from clinical samples. SVA whole genome sequence was generated using cell culture grown viruses as well as clinical samples. The methods established here, which incorporate sequencing and bioinformatics for disease diagnosis, will accelerate more precise SVA detection in the field, and with the careful curation of deposited sequences, increase the number of SVA whole genome sequences available in GenBank to improve our understanding of the virus and thus allow for better control of this disease. Additionally, this work will provide insights into the potential use of the portable MinION sequencer as a diagnostic tool to aid in the surveillance and investigation of emerging viral infectious disease in the future. 

## 2. Materials and Methods

### 2.1. SVA Samples

An SVA lab isolate (GenBank: MN164664) and clinical samples from swine SVA-positive vesicular fluid were provided by Dr. Fabio A. Vannucci at the University of Minnesota Veterinary Diagnostic Lab. The SVA lab isolate was propagated in cell culture in NCI-H1299 non-small cell lung carcinoma cell line (ATCC CRL-5803) as previously described [31]. Negative swine sera were spiked with the SVA lab isolate to generate “spike-in” samples. We tested 8 clinical samples in total, which were represented by vesicular fluid samples from SVA-positive animals.

### 2.2. RNA Extraction

SVA RNA was extracted from cell culture SVA supernatants (cell culture samples), virus-free pig serum spiked with the SVA lab isolate (spike-in samples), and clinical vesicular fluids (clinical samples) using the QIAamp Viral RNA mini kit (Qiagen, Germantown, MD, USA) following the manufacturer’s instructions without the addition of carrier RNA and with a final elution in 50 μL nuclease-free water. The concentration of the viral RNA was performed using a SpeedVac lab concentrator (Savant, NY, USA). A Qubit3.0 fluorometer (Life Technologies, Carlsbad, CA, USA) and a Nanodrop1000 spectrophotometer (Thermo Scientific, Waltham, MA, USA) were used for RNA quantity and quality assessments.

### 2.3. Oxford Nanopore Sequencing

Viral RNA was sequenced using 2 different kits, the direct RNA sequencing kit or the PCR-cDNA sequencing kit (ONT, Oxford, UK). The input RNA for the direct RNA sequencing (DRS) library preparation was isolated SVA RNA with the addition of the RNA calibration strand (RCS, 1314 bp), which was provided in the sequencing kit, to increase the amount of total input RNA which is recommended for optimized results. The RCS is Enolase II mRNA (YHR174W, NCBI Reference Sequence: NC_001140.6) provided at a concentration of approximately 50 ng/μL. Library preparation was performed according to the direct RNA sequencing online protocol (DRS_9080_v2, ONT, Oxford, UK), which includes the addition of a sequencing adaptor ligated at the 3′ end of the RNA and is used for initiation of sequencing [32]. The input RNA for the PCR-cDNA sequencing was the extracted SVA RNA only. PCR-cDNA sequencing (PCS) libraries were generated according to the PCR-cDNA sequencing online protocol (PCS_9085_v109, ONT, Oxford, UK). After library preparation, the DRS and PCS libraries were loaded onto a R9.4.1 SpotON flow cell and sequenced using a MinION Mk I sequencer (ONT, Oxford, UK) which was connected to a computer and remotely controlled by the MinKNOW software (ONT, Oxford, UK).

For the genome sequencing of the cell culture SVA lab isolate, two sequencing replicates were performed for both DRS and PCS, with the DRS starting with 60 ng SVA RNA plus 300 ng RCS and 60 ng SVA RNA alone for PCS. For the sequencing runs of clinical samples and negative pig serum spiked with the SVA lab isolate, the same amount of SVA RNA was used for the DRS and PCS library preparation, with the addition of 300 ng RCS to each of the DRS samples to increase the amount of total RNA input amounts for optimized sequencing output. Samples were sequenced for approximately 6 h and the estimated sequence yield was monitored in real time. All samples were sequenced individually. Flow cells were reused following nanopore guidelines until the number of total available pores was under 300. To minimize the potential contamination of previous runs and to protect the accuracy of the detection limit, samples with lower viral titers were sequenced first on a flow cell, followed by those with higher viral levels.

### 2.4. Bioinformatics Analysis

Basecalling was carried out using Guppy (ONT, Oxford, UK). Only raw sequencing reads that passed the quality filter of the Phred quality score ≥7 (pass reads) were used for downstream analysis. For the DRS, raw reads of RNA Control Strand (RCS, 1314 bp), which was used to enhance library preparation and sequencing performance, were filtered out by turning on the corresponding Guppy parameter. The total yield, pass read yield, read quality, and the read length of raw reads from whole genome sequencing were analyzed using MinIONQC [33], a script written in R to provide quality control for Oxford Nanopore data.

For the sequencing of cell culture samples, raw pass reads were mapped to the SVA reference genome (GenBank: MN164664) using Minimap2 [34], then analyzed using Qualimap [35], generating raw error rates and coverage information which was then visualized using GraphPad prism software (GraphPad Software, San Diego, CA, USA). The reads that mapped the SVA reference genome (SVA reads) were extracted using SAMtools [36], and the SVA yield, average read length, and quality were determined using NanoPlot [37].

For the spike-in and clinical samples, taxonomic analyses at the species level was performed to identify pathogens existing in the sample using What’s In My Pot (WIMP), which is provided by ONT’s subsidiary Metrichor [38]. An SVA custom database was created to analyze the SVA sequencing reads by downloading all of the SVA whole genomes available from GenBank (132 complete SVA genomes as of March 2019). To detect SVA at the strain level, pass reads were analyzed against this SVA database using the Basic Local Alignment Search Tool (BLAST) [39] to identify the strain with the best match based on BLAST bit score.

### 2.5. SVA Consensus Generation and Optimization

Viral consensus sequences were generated using different assemblers and the results were compared to determine the optimal assembler for each sequencing method. Four different assemblers, Canu [40], Miniasm [34], Racon [41], and wtdbg2 [42], were used to examine the reads from direct RNA sequencing and PCR-cDNA sequencing. For DRS, an optimal consensus sequence was generated, without need for reference or assembly, by extracting the longest read among all sequencing reads as a scaffold and mapping all pass reads to this longest read sequence using Minimap2 [34] followed by consensus generation using Racon [41]. For PCS, de novo assembly was performed using the Canu assembler [40]. After determining the consensus generation strategies for both sequencing methods, optimization was performed in terms of total input sequencing yield and the pre-treatment of raw reads using the cell culture virus in which the whole genome sequence was already known. Groups containing different input sequencing yields, ranging from 0.7 to 70 megabases (Mb), were generated by random selection using fastq-tools-0.8 (https://homes.cs.washington.edu/~dcjones/fastq-tools/) from the dataset of total pass reads. In the same yield group, three subgroups were formed using different raw reads filters; (1) original pass reads (Phred quality ≥7) without further filters; (2) pass reads with a read length >1314 bp to remove short reads and the RCS; and (3) pass reads that can be mapped to the SVA database. The consensus length and accuracy were the two main parameters evaluated for comparison. Consensus accuracy was determined by comparing the consensus genome to the reference genome and was analyzed using the ClustalW pairwise alignment in Geneious v8.0.5 software (https://www.geneious.com, San Diego, CA, USA) [43]. 

For the spike-in and clinical samples, a consensus sequence was able to be generated for most samples. SVA reads were first extracted by mapping all of the reads against the custom SVA database (generated above), followed by consensus generation using Racon for DRS and Canu for PCS. The consensus length and accuracy were calculated to indicate the performance of consensus generation using varying amounts of input viral copies.

### 2.6. Sanger Sequencing and Analysis

Sanger sequencing was performed for all 8 clinical samples. Primers were designed for PCR amplification of the 3′ end (3′ UTR, 3D and partial 3C genomic regions) of the SVA genome, similar to previous studies [44] (forward primer_1 5′ GGGTGACGACTTACAAGGGA 3′, reverse primer_1 5′ GAGCCAGTGCCGTGTGAAGAGT 3′; forward primer_2 5′ CGCCAAGTTTCAATCCCATC 3′, reverse primer_2 5′ TCCCTTTTCTGTTCCGACTG 3′). Samples were sequenced as previously described [45]. Basically, SVA RNA was PCR-amplified using AccuStart PCR SuperMix (Quantabio, Beverly, MA, USA) and treated with ExoSAP-IT (Thermo Fisher Scientific, Waltham, MA, USA). Sanger sequencing was performed at the University of Minnesota Genomics Center. Results and sequence quality were visualized using CLC Genomics Workbench v11.0 (https://www.qiagenbioinformatics.com/, Redwood City, CA, USA). A consensus sequence for each clinical sample was generated using the Geneious software version 8.05 (Biomatters, Auckland, New Zealand) [43]. The Sanger sequence data were submitted to the GenBank database and are available through accession numbers MN990489–MN990496, and MN997126. Sanger sequencing references are also available at Figshare (https://figshare.com/articles/Rapid_SVA_detection_using_MinION_sequencing/11728875).

### 2.7. Analytical Sensitivity Determination 

Analytical sensitivity was determined for both direct RNA sequencing and PCR-cDNA sequencing using spike-in and clinical samples containing a range of input virus amounts. For spike-in samples, an SVA viral stock was 10-fold serially diluted from 1× to 10,000× to generate decreasing amounts of virus which were then added into the SVA-free pig serum. These spike-in samples ranged from 10^2^ to 10^7^ viral copies/mL (Ct values ranging from 25 to 10). The Ct value and viral copies for all samples were determined by RT-qPCR at the University of Minnesota Veterinary Diagnostic Lab. For the clinical samples, 8 vesicular fluid clinical samples ranging from 10^2^ to 10^6^ viral copies/mL (Ct values ranging from 24 to 13) were sequenced. For both sample sets, viral RNA was extracted from 1 ml of sample with half of the sample used for direct RNA sequencing (DRS) and half for PCR-cDNA sequencing (PCS). 

For spike-in samples, the SVA strain was determined by blasting raw sequencing reads to the custom SVA database, and then the detected strain was compared to the known reference genome using ClustalW pairwise alignment from the Geneious software [43] to identify the accuracy of the strain level detection. For the clinical samples, the consensus sequence generated from Sanger sequencing was used as a partial reference genome. To obtain the whole genome reference (referred to as “reference sequence”), we performed BLASTn to get the whole genome of the strain in GenBank with the best match. A comparison was made between the strain identified as the best BLAST match of the MinION consensus sequence to the reference sequence. We then performed ClustalW pairwise alignment to compare the “best match strain for the MinION concensus” and the reference genome using Geneious v8.0.5 software (https://www.geneious.com, San Diego, CA, USA) to determine the detection accuracy [46]. To minimize the effect of contamination from previous runs on the accuracy of analytical sensitivity, samples with lower viral titers were sequenced first when using the same flow cell.

A correlation analysis was performed to test if DRS and PCS were quantitative diagnostic methods. The total number of reads varied for each sequencing reaction, thus reads were normalized by calculating the ratio of SVA reads/total reads in order to compare between samples. Linear regression analysis was then performed to determine if there was any correlation using the SVA reads/total reads ratio and the amount of input viral copies using GraphPad prism software (GraphPad Software, San Diego, CA, USA). 

### 2.8. Oxford Nanopore MinION Sequencing Data and Analysis Pipelines

The sequencing data were deposited at the NCBI Sequence Read Archive (SRA) and are available under accession numbers: SRR11124084 to SRR11124098.

Detailed information of our pipeline used for analyzing raw sequencing reads can be found at https://github.com/ShaoyuanTan/svaproject.

## 3. Results

### 3.1. Assessment of Raw Reads from Direct RNA Sequencing and PCR-cDNA Sequencing 

In order to evaluate and compare the general performance of DRS and PCS for viral whole genome recovery, two whole genome sequencing runs using a cell culture-grown virus with a known reference sequence were carried out for each method (Table 1). All runs started with 60 ng of SVA RNA for the library preparation and were sequenced for 6 h. The available pores for each sequencing run were recorded to indicate the condition of the flow cell (Table 1). SVA reads were extracted from total sequencing reads and analyzed. The PCS had a much better performance than DRS in terms of higher SVA yield (DRS 4.5Mb, PCS 66.1Mb), longer average read length (DRS 1267bp, PCS 1721bp), and lower raw error rates (DRS 15.14%, PCS 11.23%) (Table 1). These differences could be explained by the intrinsic features of Oxford Nanopore DNA sequencing (PCS) and RNA sequencing (DRS), where the latter is a novel technology still under development and DNA sequencing has been well optimized. The main reason for the higher SVA yield from the PCS would be that although the two methods started with the same amount of SVA RNA, PCS involves a PCR amplification step which increases the number of SVA DNA strands available for sequencing.

**Figure 1:**
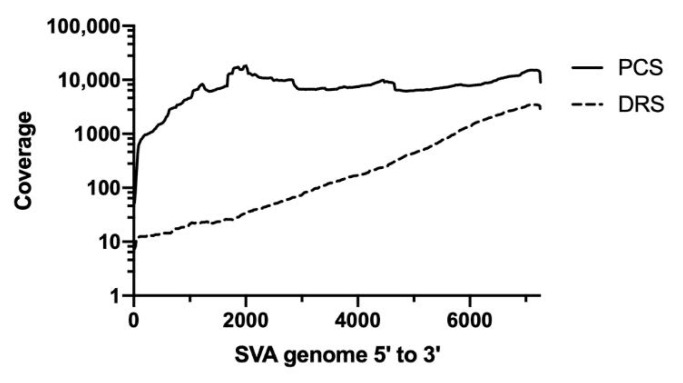
Coverage distribution of the direct RNA sequencing and PCR-cDNA sequencing. SVA reads were mapped to a reference genome using Minimap2 and analyzed by Qualimap to generate a coverage information file which was then visualized using GraphPad prism software. The dashed line represents the distribution of the direct RNA sequencing reads (DRS) and the solid black line represents the distribution of the PCR-cDNA sequencing reads (PCS).

Coverage analysis showed that PCS was able to generate a more even coverage distribution than DRS (Figure 1). For DRS, significantly more coverage was seen at the 3′ end. This uneven distribution of DRS has been observed previously and may be explained due to partially degraded RNA and RNA secondary structures hampering the movement of the RNA through the nanopores, exhibiting higher coverage where sequencing is initiated, which is at the 3′ end of the genome [47,48,49].

### 3.2. Optimization of Consensus Sequence Generation

Different assemblers were tested to determine the best fit assembler for the sequencing data from DRS and PCS. In terms of consensus length and accuracy, DRS datasets were assembled best using Racon [41], and the PCS datasets were assembled best using Canu [40]. After choosing the assembler, pre-assembly read filters were examined to determine the optimal conditions for the generation of an optimized consensus sequence. 

Datasets containing different sequencing yields (0.7, 7, and 70 Mb) were generated by randomly selecting reads from the total pass reads dataset. Within the same yield dataset, three groups were generated based on different filters, with group 1 containing all the pass reads (Phred quality >7), group 2 consisting of pass reads with a length filter >1314 bp to remove short reads and all RCS reads (RCS was added to DRS to increase the efficiency of library preparation), and group 3 with pass reads that mapped to the SVA database. The rationale behind the length filter was to test if a dataset with longer reads on average would help with consensus generation, and at the same time to delete all remaining RCS reads. Although the RCS reads should all be removed during basecalling, in fact more than a third of the RCS reads remained in the pass reads dataset due to low filtering efficiency. The rationale for the use of the mappable filter was the assumption that a “less noisy” dataset would be beneficial for SVA assembly and consensus generation, especially in some clinical samples where the desired viral RNA reads would only account for <1% of the total sequencing reads. Using the “70 Mb yield” datasets as an example, we evaluated the effect of the different filters on the read recovery, read length and read quality (Table 2). The read recovery of the DRS dataset after the length filter (group 2) was 11% of the pass reads (group 2 yield/group 1 yield) and after the SVA mappable filter (group 3) it was 7% of the pass reads (group 3 yield/group 1 yield) (Table 2). The low recovery was due to the large number of short reads present, mainly RCS reads. Read recovery of the PCS dataset after the length filter (group 2) and SVA mappable filter (group 3) was 75% and 73% of the pass reads (group 1), respectively (Table 2). The average read length and Phred quality score was greater for PCS than for DRS irrespective of the filters (Table 2). The average read length of the unfiltered DRS reads (group 1) was especially low and was mainly due to the presence of short RCS reads, which account for >90% of reads that are less than 1314 bp (Table 2). 

Examination of each read filter at different sequence yields was performed to determine the optimal conditions for the generation of a consensus sequence. For the DRS groups, as the starting yield increased, the length and accuracy of the generated consensus sequence increased (Table 3). The highest consensus length and accuracy were observed at the 70 Mb yield (~5 Mb of SVA reads, 7% SVA mappable rate) (Table 2 and Table 3). At the same yield level, the consensus accuracy and length with different filters were similar, indicating that the sequencing yield is the leading factor for consensus accuracy and length and the raw read filters have minimal influence on results (Table 3). A similar observation was observed for PCS sequencing, as within a sequencing yield, the different filters showed similar consensus length and accuracy (Table 3). However, for PCS, an increase in yield did not always result in better consensus generation, as 70 Mb pass reads generated a lower accuracy and a shorter consensus than that of the 7 Mb read group (Table 3). The most accurate consensus for PCS was generated using a total sequencing yield of 7 mb (~5 Mb of SVA reads, 73% SVA mappable rate) (Table 2 and Table 3).

While both DRS and PCS can generate a nearly full-length SVA genome, the consensus from PCS achieved a 99% accuracy, much higher than that of DRS which only reached a 94% accuracy (Table 3). In this study, no obvious differences were observed when comparing the different filters using the cell culture samples. However, the filters may be useful in some situations not examined here such as in clinical samples from tissues that would contain a large amount of host RNA. In order to make our pipeline applicable to all sample types, we used the SVA mappable reads (group 3 filter set) for the following spike-in and clinical sample analysis.

### 3.3. Determination of Analytical Sensitivity of Direct RNA and PCR-cDNA Sequencing

The analytical sensitivity of Oxford Nanopore DRS and PCS was evaluated by sequencing spike-in and clinical samples with a range of 4.7 × 10^2^ to 1.0 × 10^7^ viral copies. After sequencing, the number of total reads from each run was determined (Table 4). In order to detect SVA at the species level in an unbiased and hypothesis-free manner, the taxonomic analysis was performed using WIMP and the number of reads classified as SVA were recorded (Table 4). Results showed that SVA was able to be easily detected using both sequencing methods in both spike-in and clinical samples containing more than 5 × 10^4^ total viral copies. Using DRS to investigate spike-in samples, SVA reads were detected in samples with as low as 4.7 × 10^2^ SVA viral copies, while for PCS, SVA reads were detected in samples with viral copies of 1.2 × 10^4^ or greater. In clinical samples, the detection limit was 9.2 × 10^2^ viral copies for DRS and 2.2 × 10^3^ viral copies for PCS. The number of total reads indicated the overall performance of sequencing, while the ratio of SVA reads to total reads suggested the presence and abundance of SVA in a sample (Table 4). As a reference for future experimental design, at least 1 SVA read should be obtained if sequencing a minimum of 5 × 10^4^ viral copies from a clinical sample and generating around 10^4^ total reads (Table 4).

Of note, SVA was not detected using PCS from a clinical sample with viral copies of 1.2 × 10^4^, but was detected in other clinical samples with lower numbers of viral copies (Table 4). This could be explained by poor flowcell performance since few total reads were generated in the 1.2 × 10^4^ viral copy clinical sample. For example, even though the 1.2 × 10^4^ viral copy sample has five times greater viral copies than the 2.2 × 10^3^ viral copy sample, the total reads generated was 16 times less, thus explaining why a sample with a higher viral copy number did not detect SVA; poor sequencing performance and too few total reads generated for this sample. Our observation of varying total and SVA reads generated from samples with similar viral copies and sequencing time indicated inconsistent sequencing output for each run, mostly due to the condition of the flow cell used.

For epidemiological and precise infection control purposes, it is necessary to know not only the infectious virus present, but the strain of the virus-causing disease. Thus, to investigate whether DRS or PCS can identify the strain of SVA that is present in a sample, total reads were BLASTn analyzed against our SVA whole-genome database and the sequence with the best match (top BLAST hit based on bit score) was considered as the SVA strain present in the sample (Table 4). The percent identity between the top BLAST hit and the known sequence of the sample was determined to identify the accuracy of strain level detection (Table 4). For the spike-in samples, a laboratory strain with a known whole genome reference sequence (MN164664) was used and this sequence was also present in our SVA whole genome database. The MN164664 sequence was compared to the top BLAST hit to determine the percent identity which indicates the accuracy of strain level detection (Table 4). For each of the clinical samples, a partial genome reference sequence was obtained using Sanger sequencing, which was then used to compare with the top BLAST hit, but since these reference sequences are partial sequences, they are not present in our SVA whole-genome database, so we did not expect a 100% identity between the top BLAST hit and our reference sequence (Table 4). Both sequencing methods were 100% accurate when detecting strains for the spike-in samples, in which the reference strain was present in the SVA whole genome database and it was identified as the best match (Table 4). For clinical samples, a comparison of the known partial genome to that of the top BLAST hit showed a sequence identity of 97.0–98.2% for both sequencing methods (Table 4). Some disagreements observed between the DRS and PCS “Best match” genome revealed a limitation of detection accuracy, which can be observed between highly similar strains (Table 4).

Further examination of sequencing accuracy was determined by creating a consensus genome which was then compared to the known reference sequence to determine the sequencing accuracy. All raw reads that were mapped to the SVA database were used to generate a consensus sequence. This consensus sequence (or longest read when no consensus could be generated) was then compared to the known viral reference sequence (Table 4). A nearly complete SVA consensus genome (breadth of coverage >95%) was generated using both DRS and PCS sequencing methods from samples containing 1.1 × 10^6^ viral copies or more, giving an accuracy greater than 91% for DRS and greater than 99% for PCS (Table 4). In these experiments, a consensus genome coverage of 95% required a minimum level of 299 SVA reads for DRS and 436 SVA reads for PCS. For samples containing less than 10^6^ viral copies, a shorter consensus genome was obtained with lower accuracy (Table 4). 

Then, a quantitative relationship between the output SVA reads and the input SVA viral copies was investigated. In order to minimize the inter-sequencing variations, SVA reads were normalized for each sequencing run based on the total reads generated. A correlation analysis between the ratio of SVA reads/total reads and input SVA viral copies was performed. The results showed that DRS had a strong linear regression with an r^2^ = 0.99 while PCS had a weak linear regression with an r^2^ = 0.54, indicating that DRS was a quantitative method while PCS was not. Considering that PCS contains more steps than DRS that can introduce bias, such as PCR amplification and amplicon selection, this was not surprising.

## 4. Discussion

The early and reliable detection of infectious agents as soon as clinical signs are observed is essential for efficient disease control. Delays and misdiagnosis inevitably lead to the spread of disease and escalation of adverse impacts. Prompt actions against an emerging pathogen are especially important because there is usually no existing immunity among the susceptible population, no vaccine, and no specific treatment against the pathogen. However, emerging infections are more difficult to identify since most diagnostics are based on previously known and expected infectious agents and miss unexpected pathogens. Diagnostic methods that are rapid, available at the point-of-care, able to detect new pathogens, and robustly applicable across a wide range of pathogens are greatly needed to effectively fight against emerging eventualities [50,51]. Among all pathogens, RNA viruses have the highest mutation rates, and are anticipated to have the highest possibility to cause the next emerging event [52]. They are also of special concern regarding zoonotic transmission due to their high adaptability to new hosts [53]. In this study, we evaluated Oxford Nanopore MinION sequencing for SVA investigation, aiming to provide insights and tools for the investigation of emerging RNA viral diseases through sequencing and bioinformatics.

Oxford Nanopore provides two methodologies for RNA sequencing: traditional amplicon sequencing (PCR-cDNA sequencing, PCS), which has lower error rates and higher throughput, but involves reverse transcription and PCR amplification, which is time consuming and loses some RNA genome structure information through the process; and direct RNA sequencing (DRS), which is an innovative technique under development that can sequence RNA strands directly, thus eliminating the length limitations possibly coming from reverse transcription and allowing for the detection of nucleic acid base modifications. Both sequencing methods used in this study can be used to detect unknown RNA viral pathogens. However, a poly(A) tail, which is present in the SVA genome, is needed for adapter ligation. Thus, this approach lends itself readily to the sequencing of RNA viruses with a 3′ poly(A) tail. Many important swine RNA viral pathogens have a 3′poly(A) tail, such as coronaviruses (porcine epidemic diarrhea virus), picornaviruses (FMDV, SVA) and arteriviruses (PRRSV). The sequencing of RNA pathogens which do not contain a poly(A) tail (such as rotaviruses) can be performed through the enzymatic addition of a 3′ poly(A) tail and this step can be added to any sequencing reaction without interfering with the sequencing of samples already containing a poly(A) tail [54]. This study provided a thorough comparison between the PCS and DRS methods, which are summarized in Table 5, aiming to provide guidance on the selection of a sequencing method when in different clinical situations and for different purposes. We identified that PCS is more time consuming, but can generate a more accurate consensus, the advantage of which was especially obvious with higher viral copy number samples. Although DRS was observed to be less accurate, it was quicker to perform and just as sensitive and has unique and promising features such as the detection of nucleic acid modifications, as observed by other studies [55,56]. Despite their differences, both sequencing methods were able to accurately detect SVA at the strain level using raw reads and entry-level bioinformatics analysis (Table 5). Thus, a core sequencing laboratory with data analysis experts was not necessary for the detection of the strain of SVA present, suggesting it could be run on a farm or at least more quickly than other more analysis-intense sequencing methods.

The analytical sensitivity of a diagnostic method gives important information to help guide method selection based upon the situation. The evaluation of the analytical sensitivity of MinION sequencing requires the definition of either sequencing time or the minimum number of total reads. In this study, the idea of a same day report was desired, so a rapid turnaround time frame using only 6 h of sequencing was performed. The analytical sensitivity for both DRS and PCS was shown to be similar with an input of 5 × 10^4^ viral copies or more (in 0.5 mL starting material) always generating SVA reads. DRS was slightly more sensitive at approximately 10^2^–10^3^ viral copies (per 0.5 mL), while PCS needed approximately 10^3^−10^4^ viral copies (per 0.5 mL) to detect SVA. Previously, it had been shown that the MinION sequencing of influenza virus had a detection limit of 10^2^–10^3^ genome copies/mL for 48 h of sequencing, showing a similar sensitivity to our DRS experiments [57]. Similar to other studies, we observed a great inconsistency between runs within the same sequencing time frame, which could be caused by factors such as varying flow cell conditions and sample quality [58]. Using the number of sequencing reads as well as sequencing time (monitored in real time using the MinKNOW interface) can help minimize this sequencing run to run variation. In fact, we were able to determine that an input of more than 5 × 10^4^ viral copies from a clinical sample and obtaining around 10^4^ total sequence reads were needed to generate a minimum of one SVA read. If more SVA reads were desired for other purposes, such as whole genome generation, or if a lower amount of sample was used, then more total reads should be set as a target.

From this study, an advantage of direct RNA sequencing over amplicon sequencing, such as PCR-cDNA, was that DRS showed a quantitative relationship between input viral titers and output sequencing reads. Similarly, a strong relationship between influenza viral titers and influenza sequencing reads using Oxford Nanopore direct RNA sequencing technology was observed by other research groups [57]. However, in a hepatitis B virus (HBV) study using Oxford Nanopore amplicon sequencing (which includes a PCR step similar to our PCS protocol), considerable variability in total yields and the proportion of mapped HBV reads between sequencing runs was observed concluding that it was not quantitative [59]. Amplicon sequencing, such as the PCR-cDNA protocol, includes more steps during library preparation, including the amplification and selection of PCR products which could possibly introduce bias, while the process of direct RNA library preparation is simple and straightforward without additional amplification steps.

While most sequencing is generally restricted to large laboratories, the portability of the MinION sequencer makes it suitable for diagnosis in the field. On-site diagnosis can greatly improve emerging infectious disease management, especially considering that emerging disease outbreaks can happen anywhere and are more likely to occur in developing countries or remote areas where there is a lack of veterinary infrastructure, expertise, and diagnostic capacities [4,60]. In fact, several field studies have been conducted to confirm such advantages of a portable sequencer including a Zika virus outbreak in Brazil, a 2015 Ebola outbreak, and a Dengue virus field investigation [28,61,62], concluding that the use of the MinION sequencer was advantageous for rapid in-field disease detection. 

There are a few limitations to using these sequencing methods for emerging disease detection. First, our method of species and strain detection largely depends on the genome database, GenBank. While the examination of emerging viral diseases caused by known viruses expanding to new hosts or geographical regions will have viral genome information available in GenBank, previously unknown or newly discovered pathogens will not. However, the sequencing information for these unknown or newly discovered pathogens can be determined following MinION sequencing by carefully examining the unclassified sequencing reads. Second, the analytical sensitivity of MinION sequencing is lower than that of diagnostic PCR assays, but PCR assays are limited to the detection of known pathogens and during an outbreak, high levels of the pathogen should be present allowing for the ease of detection through MinION sequencing. In addition, sequencing, even at the current sensitivity of detection, in the case of new pathogens can be used to support PCR by providing strain information for more effective disease control and for epidemiologic studies to track infection. Third, while this study provided a benchmark and foundation for portable sequencer use in disease diagnostics, there is still more to do to achieve commercial diagnostics, such as the improvement of the accuracy, detection limit and consistency of flow cell performance. 

This study evaluated the ability of MinION sequencing for use as a diagnostic tool for the detection of emerging viral diseases in swine by examining SVA infection as a model of an emerging disease. We demonstrated that the portability, easy-operation, low-maintenance MinION platform is an effective tool for the investigation of SVA. We provided a detailed pipeline of our analysis of raw reads to help investigators use this technology (https://github.com/ShaoyuanTan/svaproject). The methods established in this study provide a framework for prompt diagnostics of other emerging viral diseases. Infectious diseases will continue to emerge around the world and it is increasingly important to be prepared for the next outbreak [63]. 

## Figures and Tables

**Table 1:** A comparison between the sequencing statistics of the direct RNA and PCR-cDNA methods after 6 h sequencing *.

Sequencing Statistics	Direct RNA Sequencing (DRS)	PCR-cDNA Sequencing (PCS)
Number of available pores	403 ± 100	421 ± 18
Total pass yield (Mb)	68 ± 0	92 ± 24
Senecavirus A (SVA) yield (Mb)	4.5 ± 0.6	66.1 ± 16.2
Number of SVA reads	3559 ± 358	38,544 ± 0
Mean SVA read length	1267 ± 40	1721 ± 47
SVA read error rate (%)	15.14 ± 0.32	11.23 ± 0.23

* Data shown as the mean ± SD of 2 independent replicates.

**Table 2:** Sequencing raw read statistics after using different read filters *.

Groups	Sequencing Statistics	Direct RNA Sequencing (DRS)	PCR-cDNA Sequencing (PCS)
Group 1	Yield (Mb)	68 ± 0	70 ± 0
Read quality > 7	Average length (bp)	621 ± 2	1506 ± 19
	Average quality	8.5 ± 0.4	8.6 ± 0.1
Group 2	Yield (Mb)	7.2 ± 0.4	52.6 ± 0.8
Read quality >7+	Average length (bp)	2314 ± 35	2977 ± 24
length > 1314 bp	Average quality	8.6 ± 0	8.9 ± 0
Group 3	Yield (Mb)	4.5 ± 0.6	66.1 ± 0.8
Read quality >7+	Average length (bp)	1267 ± 40	1726 ± 49
mapped to SVA database	Average quality	8.3 ± 0.1	8.6 ± 0.1

* Data shown as the mean ± SD of 2 independent replicates.

**Table 3:** Performance of consensus generation using different raw read filters at different yields *.

		Group 1		Group 2 (Length Filter)		Group 3 (SVA Mapped)		
Sequencing method	Yield (Mb)	Consensus length (bp)	Accuracy (%)	Consensus length (bp)	Accuracy (%)	Consensus length (bp)	Accuracy (%)	SVA reads ^^^
DRS	0.7	5098 ± 786	89.3 ± 2.4	4881 ± 1728	89.8 ± 1.8	6057 ± 1143	86.2 ± 3.5	55 ± 2
	7	7155 ± 4	90.8 ± 1.5	5522 ± 1229	91.2 ± 3	7091 ± 45	90.8 ± 1.3	548 ± 20
	70	7163 ± 18	94.3 ± 0.2	7096 ± 30	94.4 ± 0.4	7110 ± 21	94.4 ± 0.5	3559 ± 358
PCS	0.7	6738 ± 424	99 ± 0.1	6316 ± 541	97.4 ± 2.4	6592 ± 489	98 ± 1.0	410 ± 6
	7	7267 ± 132	98.9 ± 0.4	7238 ± 19	99.0 ± 0.1	7079 ± 161	99.0 ± 0.0	4092 ± 64
	70	3053 ± 35	90.5 ± 11.5	6596 ± 1903	91.6 ± 5.4	6761 ± 995	87.9 ± 2.0	38544 ± 10439

* Data shown as the mean ± SD of 2 independent replicates; the whole genome length of this SVA strain is 7268 bp. ^^^ Number of reads mapped to SVA, only determined for group 3.

**Table 4:** Analytical sensitivity of direct RNA sequencing (DRS) and PCR-cDNA sequencing (PCS).

					Species Detection			Strain Level Identification				Consensus Generation					
	Ct value	Viral copies	Total reads		WIMP ^‡^ SVA reads		GenBank reference sequence *	DRS		PCS		Minimap SVA reads		DRS consensus		PCS consensus	
			DRS	PCS	DRS	PCS		Best match	Identity (%)	Best match	Identity (%)	DRS	PCS	Length ^^^ (bp)	Accuracy (%)	Length ^^^ (bp)	Accuracy (%)
Spike-in samples	10	1.0 × 10^7^	94,572	16,515	5722	2913	MN164664	MN164664	100.0	MN164664	100.0	42,936	4658	7189	94.8	7395	99.2
	13	1.2 × 10^6^	40,555	5775	236	1182	MN164664	MN164664	100.0	MN164664	100.0	1961	1611	7177	91.0	6873	99.0
	18	6.5 × 10^4^	185,259	5071	17	296	MN164664	MN164664	100.0	MN164664	100.0	43	420	2551	90.6	6770	99.4
	20	1.2 × 10^4^	219,789	3889	3	3	MN164664	MN164664	100.0	MN164664	100.0	10	3	1306	90.3	916 ^†^	80.8
	25	4.7 × 10^2^	110,641	7880	1	0	MN164664	MN164664	100.0	NA	NA	1	0	456 ^†^	85.1	NA	NA
Clinical Samples	13	1.1 × 10^6^	45,478	78,075	299	436	MN990489	KX019804.1	97.1	KX019804.1	97.1	377	630	7157	97.0	7013	99.6
	16	1.3 × 10^5^	148,513	5256	83	7	MN990490	KX019804.1	98.2	KU058182.1	97.8	124	9	3421	96.4	880 ^†^	89.0
	18	5.0 × 10^4^	47,411	174,551	19	24	MN990491	KY618836.1	97.4	KY618836.1	97.4	20	27	6534	97.1	2285 ^†^	95.3
	20	1.2 × 10^4^	54,964	5943	3	0	MN990492	KU051394.1	97.9	NA		4	0	1957	82.4	NA	NA
	21	7.6 × 10^3^	39,465	242,239	3	4	MN990493	KY618835.1	97.7	MK256736.1	97.4	3	5	1158	94.3	988 ^†^	87.0
	22	2.3 × 10^3^	53,359	96,661	1	4	MN990494	KY618836.1	97.4	KT827250.1	97.7	1	7	511 ^†^	88.8	7745 ^†^	85.0
	23	2.2 × 10^3^	58,929	633,632	4	1	MN990495 MN997126	KX019804.1	98.2	MH634514.1	98.2	4	1	3206	92.1	1171 ^†^	94.5
	24	9.2 × 10^2^	41,645	135,552	1	0	MN990496	MH490944.1	97.0	NA	NA	1	0	300 ^†^	80.0	NA	NA

* Obtained from Sanger sequencing and deposited in Genbank, ^†^ Longest raw read, due to lack of consensus, ^^^ SVA whole genome is around 7.3kb, ^‡^ What’s in my pot.

**Table 5:** Summary of direct RNA sequencing and PCR-cDNA sequencing.

	Direct RNA Sequencing (DRS)	PCR-cDNA Sequencing (PCS)
Laboratory time (sample prep)	3 h	5 h
Sequencing time	6 h	6 h
Amount of RNA recommended for input	500 ng *	2 ng
Analytical sensitivity (viral copies)	10^2^ to 10^3^	10^3^ to 10^4^
Recommended consensus generation program	Racon	Canu
Raw read accuracy	85%	89%
Consensus accuracy	94%	99%
Consensus genome coverage ^^^	100%	100%
Read distribution	Coverage biases	Even coverage
Key attributes	Rapid, sensitive, potential RNA structure detection	Accurate
Key concerns	High input RNA amount *, higher error rate	Need for amplification, longer time to results

* Exogenous RNA can be used to increase RNA input requirements allowing for very low amounts (i.e., <0.02 ng) of desired RNA as input. This would then require bioinformatic analysis to separate the exogenous RNA reads from the desired reads. ^^^ Consensus genome coverage of 95% requires a minimum level of 299 SVA reads for DRS and 436 SVA reads for PCS, based on the analysis of clinical samples (Table 4).

## References

[1] Meng X.J. (2012). Emerging and Re-Emerging Swine Viruses. Transbound. Emerg. Dis..

[2] Devaux C.A. (2012). Emerging and re-emerging viruses: A global challenge illustrated by Chikungunya virus outbreaks. World J. Virol..

[3] Burrell C.J., Howard C.R., Murphy F.A., Burrell C.J., Howard C.R., Murphy F.A. (2017). Chapter 15-Emerging Virus Diseases. Fenner and White’s Medical Virology.

[4] Jones G., Patel N., Levy M., Storeygard A., Balk D., Gittleman J.L., Daszak P. (2008). Global trends in emerging infectious diseases. Nature.

[5] Lunney J.K., Benfield D.A., Rowland R.R. (2010). Porcine reproductive and respiratory syndrome virus: An update on an emerging and re-emerging viral disease of swine. Virus Res..

[6] Segalés J., Kekarainen T., Cortey M. (2013). The natural history of porcine circovirus type 2: From an inoffensive virus to a devastating swine disease?. Veter Microbiol..

[7] Goede D., Morrison R.B. (2016). Production impact & time to stability in sow herds infected with porcine epidemic diarrhea virus (PEDV). Prev. Veter Med..

[8] Jurado C., Mur L., Aguirreburualde M.S.P., Cadenas-Fernández E., Martínez-López B., Sánchez-Vizcaíno J.M., Perez A. (2019). Risk of African swine fever virus introduction into the United States through smuggling of pork in air passenger luggage. Sci. Rep..

[9] Hales L.M., Knowles N.J., Reddy P.S., Xu L., Hay C., Hallenbeck P.L. (2008). Complete genome sequence analysis of Seneca Valley virus-001, a novel oncolytic picornavirus. J. Gen. Virol..

[10] Venkataraman S., Reddy S.P., Loo J., Idamakanti N., Hallenbeck P.L., Reddy V.S. (2008). Structure of Seneca Valley Virus-001: An oncolytic picornavirus representing a new genus. Structure.

[11] Burke M.J. (2016). Oncolytic Seneca Valley Virus: Past perspectives and future directions. Oncolytic Virotherapy.

[12] Leme R.D.A., Zotti E., Alcântara B.K., Oliveira M.V., Freitas L.A., Alfieri A.F., Alfieri A.A. (2015). Senecavirus A: An Emerging Vesicular Infection in Brazilian Pig Herds. Transbound. Emerg. Dis..

[13] Wu Q., Zhao X., Chen Y., He X., Zhang G., Ma J. (2016). Complete Genome Sequence of Seneca Valley Virus CH-01-2015 Identified in China. Genome Announc..

[14] Canning P., Canon A., Bates J.L., Gerardy K., Linhares D.C.L., Piñeyro P.E., Schwartz K.J., Yoon K.J., Rademacher C.J., Holtkamp D. (2016). Neonatal Mortality, Vesicular Lesions and Lameness Associated with Senecavirus A in a U.S. Sow Farm. Transbound. Emerg. Dis..

[15] Saeng-Chuto K., Rodtian P., Temeeyasen G., Wegner M., Nilubol D. (2017). The first detection of Senecavirus A in pigs in Thailand, 2016. Transbound. Emerg. Dis..

[16] Segalés J., Barcellos D., Alfieri A., Burrough E., Marthaler D. (2017). Senecavirus A: An Emerging Pathogen Causing Vesicular Disease and Mortality in Pigs?. Vet. Pathol..

[17] Oliveira T.E.S., Michelazzo M.M.Z., Fernandes T., De Oliveira A.G., Leme R.D.A., Alfieri A.F., Alfieri A.A., Headley S. (2017). Histopathological, immunohistochemical, and ultrastructural evidence of spontaneous Senecavirus A-induced lesions at the choroid plexus of newborn piglets. Sci. Rep..

[18] Dvorak C.M.T., Akkutay-Yoldar Z., Stone S.R., Tousignant S.J.P., Vannucci F., Murtaugh M.P. (2017). An indirect enzyme-linked immunosorbent assay for the identification of antibodies to Senecavirus A in swine. BMC Veter Res..

[19] Goolia M., Vannucci F., Patnayak D., Nfon C., Yang M., Babiuk S. (2017). Validation of a competitive ELISA and a virus neutralization test for the detection and confirmation of antibodies to Senecavirus A in swine sera. J. Veter Diagn. Investig..

[20] Fowler V.L., Ransburgh R.H., Poulsen E.G., Wadsworth J., King D.P., Mioulet V., Knowles N.J., Williamson S., Liu X., Anderson G.A. (2017). Development of a novel real-time RT-PCR assay to detect Seneca Valley virus-1 associated with emerging cases of vesicular disease in pigs. J. Virol. Methods.

[21] Feronato C., Leme R.D.A., Diniz J.A., Agnol A.M.D., Alfieri A.F., Alfieri A.F. (2017). Development and evaluation of a nested-PCR assay for Senecavirus A diagnosis. Trop. Anim. Health Prod..

[22] Zhang Z., Zhang Y., Lin X., Chen Z., Wu S. (2019). Development of a novel reverse transcription droplet digital PCR assay for the sensitive detection of Senecavirus A. Transbound. Emerg. Dis..

[23] Huang B., Jennison A., Whiley D., McMahon J., Hewitson G., Graham R., De Jong A., Warrilow D., Jennsion A. (2019). Illumina sequencing of clinical samples for virus detection in a public health laboratory. Sci. Rep..

[24] Qian S., Fan W., Qian P., Chen H., Li X.-M. (2016). Isolation and full-genome sequencing of Seneca Valley virus in piglets from China, 2016. Virol. J..

[25] Zhao X., Wu Q., Bai Y., Chen G., Zhou L., Wu Z., Li Y., Zhou W., Yang H., Ma J. (2017). Phylogenetic and genome analysis of seven senecavirus A isolates in China. Transbound. Emerg. Dis..

[26] Loman N.J., Watson M. (2015). Successful test launch for nanopore sequencing. Nat. Methods.

[27] Jain M., Olsen H.E., Paten B., Akeson M. (2016). The Oxford Nanopore MinION: Delivery of nanopore sequencing to the genomics community. Genome Biol..

[28] Quick J., Loman N.J., Duraffour S., Simpson J.T., Severi E., Cowley L.A., Bore J.A., Koundouno R., Dudas G., Mikhail A. (2016). Real-time, portable genome sequencing for Ebola surveillance. Nature.

[29] Rambo-Martin B.L., Keller M.W., Wilson M.M., Nolting J.M., Anderson T.K., Vincent A.L., Bagal U., Jang Y., Neuhaus E.B., Davis C.T. (2020). Influenza A virus field surveillance at a swine-human interface. mSphere.

[30] Quick J., Grubaugh N.D., Pullan S.T., Claro I.M., Smith A.D., Gangavarapu K., Oliveira G., Robles-Sikisaka R., Rogers T.F., Beutler N. (2017). Multiplex PCR method for MinION and Illumina sequencing of Zika and other virus genomes directly from clinical samples. Nat. Protoc..

[31] Joshi L.R., Mohr K.A., Clement T., Hain K.S., Myers B., Yaros J., Nelson E.A., Christopher-Hennings J., Gava D., Schaefer R. (2016). Detection of the emerging picornavirus Senecavirus A in pigs, mice, and houseflies. J. Clin. Microbiol..

[32] Garalde D.R., Snell E.A., Jachimowicz D., Sipos B., Lloyd J.H., Bruce M., Pantic N., Admassu T., James P., Warland A. (2018). Highly parallel direct RNA sequencing on an array of nanopores. Nat. Methods.

[33] Lanfear R., Schalamun M., Kainer D., Wang W., Schwessinger B. (2019). MinIONQC: Fast and simple quality control for MinION sequencing data. Bioinformatics.

[34] Li H. (2016). Minimap and miniasm: Fast mapping and de novo assembly for noisy long sequences. Bioinformatics.

[35] Okonechnikov K., Conesa A., García-Alcalde F. (2016). Qualimap 2: Advanced multi-sample quality control for high-throughput sequencing data. Bioinformatics.

[36] Li H., Handsaker B., Wysoker A., Fennell T., Ruan J., Homer N., Marth G., Abecasis G., Durbin R. (2009). The Sequence Alignment/Map format and SAMtools. Bioinformatics.

[37] De Coster W., D’Hert S., Schultz D.T., Cruts M., Van Broeckhoven C. (2018). NanoPack: Visualizing and processing long-read sequencing data. Bioinformatics.

[38] Juul S., Izquierdo F., Hurst A., Dai X., Wright A., Kulesha E., Pettett R., Turner D.J. (2015). What’s in my pot? Real-time species identification on the MinION™. bioRxiv.

[39] Altschul S.F., Gish W., Miller W., Myers E.W., Lipman D.J. (1990). Basic local alignment search tool. J. Mol. Biol..

[40] Koren S., Walenz B.P., Berlin K., Miller J.R., Bergman N.H., Phillippy A.M. (2017). Canu: Scalable and accurate long-read assembly via adaptive k-mer weighting and repeat separation. Genome Res..

[41] Vaser R., Sovic I., Nagarajan N., Sikic M. (2017). Fast and accurate de novo genome assembly from long uncorrected reads. Genome Res..

[42] Ruan J., Li H. (2020). Fast and accurate long-read assembly with wtdbg2. Nat. Methods.

[43] Kearse M., Moir R., Wilson A., Stones-Havas S., Cheung M., Sturrock S., Buxton S., Cooper A., Markowitz S., Duran C. (2012). Geneious Basic: An integrated and extendable desktop software platform for the organization and analysis of sequence data. Bioinformatics.

[44] Wu Q., Zhao X., Bai Y., Sun B., Xie Q., Ma J. (2017). The First Identification and Complete Genome of Senecavirus A Affecting Pig with Idiopathic Vesicular Disease in China. Transbound. Emerg. Dis..

[45] Dvorak C.M.T., Lilla M.P., Baker S.R., Murtaugh M.P. (2013). Multiple routes of porcine circovirus type 2 transmission to piglets in the presence of maternal immunity. Vet. Microbiol..

[46] Larkin M., Blackshields G., Brown N.P., Chenna R., Mcgettigan P., McWilliam H., Valentin F., Wallace I., Wilm A., López R. (2007). Clustal W and Clustal X version 2.0. Bioinformatics.

[47] Tan S., Dvorak C.M.T., Murtaugh M.P. (2019). Rapid, Unbiased PRRSV Strain Detection Using MinION Direct RNA Sequencing and Bioinformatics Tools. Viruses.

[48] Keller M.W., Rambo-Martin B.L., Wilson M.M., Ridenour C.A., Shepard S.S., Stark T.J., Neuhaus E.B., Dugan V.G., Wentworth D.E., Barnes J. (2018). Direct RNA Sequencing of the Coding Complete Influenza A Virus Genome. Sci. Rep..

[49] Witwer C., Rauscher S., Hofacker I.L., Stadler P.F. (2001). Conserved RNA secondary structures in Picornaviridae genomes. Nucleic Acids Res..

[50] Caliendo A.M., Gilbert D.N., Ginocchio C.C., Hanson K.E., May L., Quinn T.C., Tenover F.C., Alland D., Blaschke A.J., Bonomo R.A. (2013). Better tests, better care: Improved diagnostics for infectious diseases. Clin. Infect. Dis..

[51] Blaschke A.J., Hersh A.L., Beekmann S.E., Ince D., Polgreen L.A., Hanson K.E. (2015). Unmet Diagnostic Needs in Infectious Disease. Diagn. Microbiol. Infect. Dis..

[52] Woolhouse M.E., Brierley L. (2018). Epidemiological characteristics of human-infective RNA viruses. Sci. Data.

[53] Carrasco-Hernandez R., Jácome R., López-Vidal Y., De León S.P. (2017). Are RNA viruses candidate agents for the next global pandemic? A review. ILAR J..

[54] Wongsurawat T., Jenjaroenpun P., Taylor M.K., Lee J., Tolardo A.L., Parvathareddy J., Kandel S., Wadley T.D., Kaewnapan B., Athipanyasilp N. (2019). Rapid Sequencing of Multiple RNA Viruses in Their Native Form. Front. Microbiol..

[55] Ovcharenko A., Rentmeister A. (2018). Emerging approaches for detection of methylation sites in RNA. Open Biol..

[56] Viehweger A., Krautwurst S., Lamkiewicz K., Madhugiri R., Ziebuhr J., Hölzer M., Marz M. (2019). Direct RNA nanopore sequencing of full-length coronavirus genomes provides novel insights into structural variants and enables modification analysis. Genome Res..

[57] Lewandowski K., Xu Y., Pullan S.T., Lumley S.F., Foster D., Sanderson N., Vaughan A., Morgan M., Bright N., Kavanagh J. (2019). Metagenomic Nanopore sequencing of influenza virus direct from clinical respiratory samples. J. Clin. Microbiol..

[58] Lu H., Giordano F., Ning Z. (2016). Oxford Nanopore MinION Sequencing and Genome Assembly. Genom. Proteom. Bioinform..

[59] McNaughton A.L., Roberts H.E., Bonsall D., De Cesare M., Mokaya J., Lumley S.F., Golubchik T., Piazza P., Martin J.B., De Lara C. (2019). Illumina and Nanopore methods for whole genome sequencing of hepatitis B virus (HBV). Sci. Rep..

[60] Davies P.R. (2012). One world, one health: The threat of emerging swine diseases. A North American perspective. Transbound. Emerg. Dis..

[61] Faria N.R., Sabino E.C., Nunes M.R.T., Alcantara L.C.J., Loman N.J., Pybus O.G. (2016). Mobile real-time surveillance of Zika virus in Brazil. Genome Med..

[62] Yamagishi J., Runtuwene L.R., Hayashida K., Mongan A.E., Thi L.A.N., Thuy L.N., Nhat C.N., Limkittikul K., Sirivichayakul C., Sathirapongsasuti N. (2017). Serotyping dengue virus with isothermal amplification and a portable sequencer. Sci. Rep..

[63] Morens D.M., Fauci A.S. (2013). Emerging infectious diseases: Threats to human health and global stability. PLoS Pathog..

